# The oDGal Mouse: A Novel, Physiologically Relevant Rodent Model of Sporadic Alzheimer’s Disease

**DOI:** 10.3390/ijms24086953

**Published:** 2023-04-09

**Authors:** Wayne Chadwick, Stuart Maudsley, William Hull, Enes Havolli, Eugene Boshoff, Mark D. W. Hill, Pascal J. D. Goetghebeur, David C. Harrison, Sohaib Nizami, David C. Bedford, Gareth Coope, Katia Real, Christoph Thiemermann, Peter Maycox, Mark Carlton, Sarah L. Cole

**Affiliations:** 1 Takeda Cambridge, 418 Cambridge Science Park, Cambridge CB4 0PZ, UK; 2 Receptor Biology Lab, University of Antwerp, 2000 Antwerp, Belgium; 3 William Harvey Research Institute, Barts and the London School of Medicine and Dentistry, Centre for Translational Medicine and Therapeutics, Queen Mary University of London, London E1 4NS, UK

**Keywords:** sporadic Alzheimer’s disease, D-galactose, cognitive deficits, neurodegeneration, advanced glycation end products, oxidative stress, amyloid beta

## Abstract

Sporadic Alzheimer’s disease (sAD) represents a serious and growing worldwide economic and healthcare burden. Almost 95% of current AD patients are associated with sAD as opposed to patients presenting with well-characterized genetic mutations that lead to AD predisposition, i.e., familial AD (fAD). Presently, the use of transgenic (*Tg*) animals overexpressing human versions of these causative fAD genes represents the dominant research model for AD therapeutic development. As significant differences in etiology exist between sAD and fAD, it is perhaps more appropriate to develop novel, more sAD-reminiscent experimental models that would expedite the discovery of effective therapies for the majority of AD patients. Here we present the oDGal mouse model, a novel model of sAD that displays a range of AD-like pathologies as well as multiple cognitive deficits reminiscent of AD symptomology. Hippocampal cognitive impairment and pathology were delayed with N-acetyl-cysteine (NaC) treatment, which strongly suggests that reactive oxygen species (ROS) are the drivers of downstream pathologies such as elevated amyloid beta and hyperphosphorylated tau. These features demonstrate a desired pathophenotype that distinguishes our model from current transgenic rodent AD models. A preclinical model that presents a phenotype of non-genetic AD-like pathologies and cognitive deficits would benefit the sAD field, particularly when translating therapeutics from the preclinical to the clinical phase.

## 1. Introduction

sAD is characterized by a progressive loss of cognitive function precipitating from multiple pathologies and is fast becoming an economic burden worldwide. The etiology of sAD is the result of multifaceted interactions among numerous genetic, epigenetic, proteostatic, and environmental factors [1,2,3,4,5,6,7]. Approximately 95% of AD patients develop the disease in a sporadic manner, while the remaining cases are associated with familial genetic mutations [2]. Multiple disease pre-disposing genes responsible for the less common fAD are known and have been utilized to produce *Tg* animal models [8]. Although these models are valuable tools for investigating the pathological mechanisms of AD, they all share a common flaw in that their etiology is representative of fAD, with pathology driven by mutant protein expression, which allows significant pathologies to precipitate in adolescence and thus may not best reproduce the etiopathology of sAD.

Clinical success for AD treatment is dependent on early pharmacological intervention [9]. In recent years however, multiple large-scale AD therapeutic efforts have failed, in part potentially due to the pre-clinical insufficiencies of standard *Tg* model-based pipelines [10,11,12,13]. Given the lack of genetic mutation correlation with the majority of patients with AD, perhaps the most pragmatic way forward to improve drug discovery and development is the creation of effective AD model organisms that do not rely on this standard approach. Certain groups have attempted to address this issue by administering Aβ [14] or streptozotocin (STZ) [15] directly into the brains of rodents to induce AD-related pathology. Although these Aβ and STZ models display AD-like pathologies and cognitive deficits, they require intricate and time-consuming surgical interventions. The Aβ model shares similarities with certain *Tg* models in that the pathologies precipitate as a result of excessive toxic Aβ build up. These data thus question the validity of whether this is a true sAD model, as it does not address the physiological processes that are responsible for this initial rise of Aβ in sAD [16,17]. The STZ model attempts to address this issue by negatively affecting energy homeostasis in the brain, which leads to AD-like pathologies. Although this model displays elevated hyperphosphorylated tau (p-tau), oxidative stress, and neuroinflammation, Aβ accumulation is sparse, and the pathology initiates in the cortex before spreading to the hippocampus [18,19,20]. This contradicts clinical observations, where pathologies typically originate in hippocampal regions before spreading throughout the brain [18,20]. The limited Aβ accumulation and cortical seeding of pathologies question the utility of this approach to modeling sAD. A murine model that better represents sAD is a critical requirement to enable successful translation of preclinical drug discovery to clinical research.

We report here on the development of a novel murine model, the oDGal model, which recapitulates behavioral alterations as well as a host of pathologies that typify general AD pathologies. Chronic D-galactose administration via intraperitoneal injection has previously been shown to accelerate natural aging in certain mouse strains [21]. D-galactose is a physiological nutrient catabolized to produce UDP-galactose, a substrate for AGE (advanced glycation end-products). AGE accumulation, a common feature of aging, is elevated in many peripheral and neurodegenerative diseases and is predicted to accelerate oxidative damage [22,23,24]. Here, we validate and optimize chronic oDGal administration via a less invasive route, i.e., drinking water, using C57Bl6/j mice, a favored mouse strain in the neuroscience community. Employment of this paradigm facilitates the creation of multiple AD-relevant features that appear to seed and spread in a similar pattern to that observed in clinical AD cases. The novel oral dosing regimen makes the model simple and affordable to run. Importantly, with respect to future drug discovery efforts, the oDGal-induced cognitive deficit was reversed with drugs currently prescribed for, or in trial for, sAD. Moreover, the cognitive decline and onset of pathology were delayed following antioxidant treatment, which strongly implies ROS as the mechanistic driver of downstream pathologies. This feature further reinforces how well the model aligns with more recent data concerning the etiology of sAD [25,26,27] as opposed to genetically-induced fAD.

## 2. Results

### 2.1. oDGal Induces Neuronal Hypometabolism

**Figure 1:**
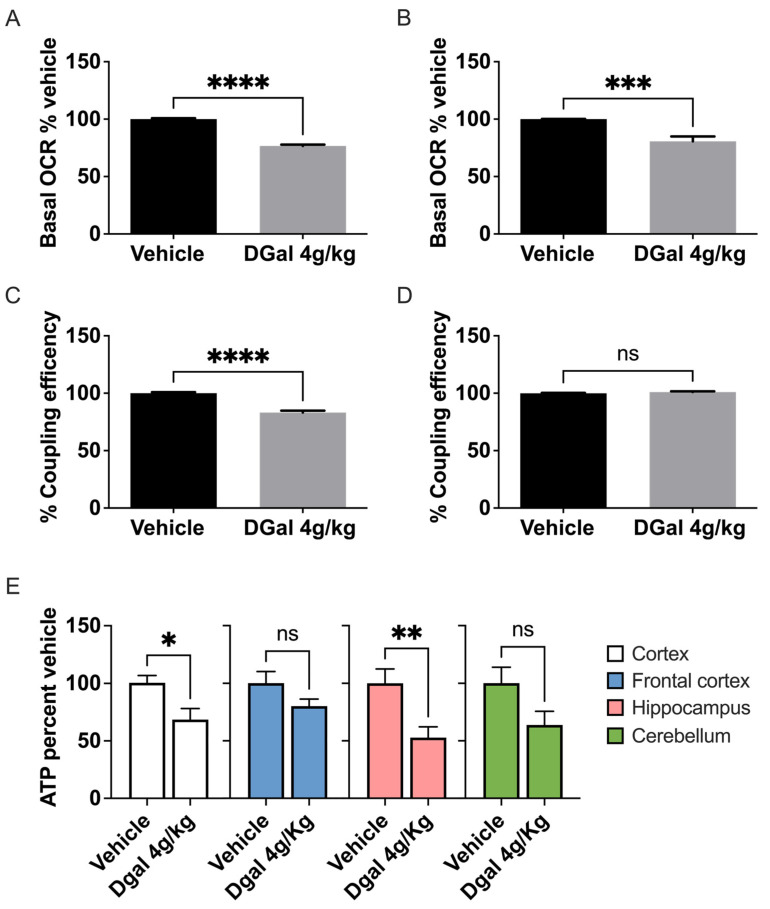
oDGal induces brain hypometabolism. (**A**) Hippocampal and (**B**) cortical basal mitochondrial oxygen consumption rate measured in synaptosomes isolated from mice dosed chronically over 8 weeks with vehicle or 4 g/kg oDGal. (**C**) Hippocampal and (**D**) cortical ATP production rates measured in synaptosomes isolated from mice dosed chronically over 8 weeks with vehicle or 4 g/kg oDGal. (**E**) Representation of ATP content in cortex, frontal cortex, hippocampus, and cerebellar homogenate fractions for mice dosed chronically over 8 weeks with vehicle or 4 g/kg oDGal. Data are presented as a mean ± SEM percentage vehicle, unpaired two-tailed student *t*-test, Asterix (*) represent significant changes: **** *p* < 0.0001, *** *p* = 0.0002, ** *p* = 0.0075, * *p* = 0.032; ns = non-significant, *n* = 10 vehicle and *n* = 10 oDGal mice.

Brain hypometabolism has long been linked with AD pathology and has become a useful tool for predicting AD progression in the clinic [28,29]. To investigate neuron-specific mitochondrial integrity, synaptosomes were isolated following 8 weeks of 4 g/kg oDGal treatment for analysis on a Seahorse. We see from these data that oDGal significantly impairs basal mitochondrial function in both the hippocampal (Figure 1A) and the cortical synaptosomes (Figure 1B). ATP production is significantly impaired in the hippocampal (Figure 1C) but not the cortical (Figure 1D) synaptosomes. We do, however, see a significant decrease in ATP from whole tissue lysate obtained from the frontal cortex, hippocampus, cortex, and cerebellum after 8 weeks of 4 g/kg oDGal treatment (Figure 1E). The dramatic reduction in ATP in whole tissue lysate indicates that oDGal treatment affects the energy homeostasis of various supporting cells in the brain more severely than neurons.

### 2.2. oDGal Increases AGE Products and Oxidative Stress

**Figure 2:**
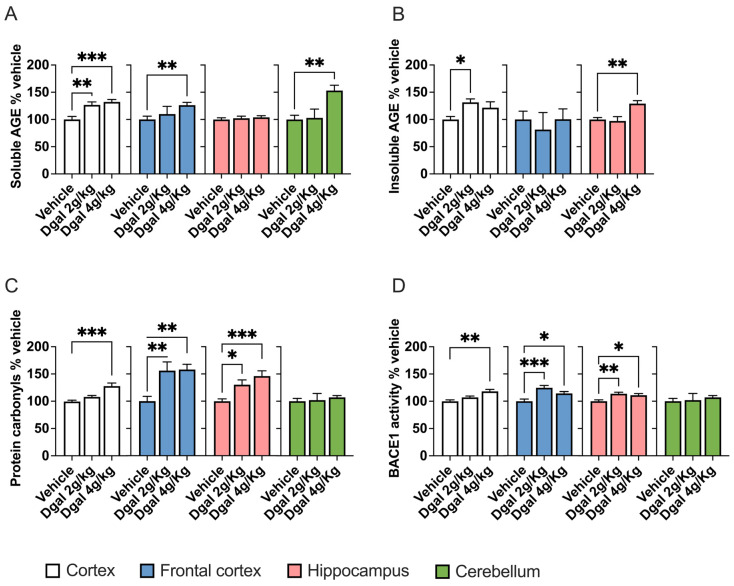
Chronic oDGal treatment elevates AGE, ROS, and BACE1 activity in the brain. (**A**) AGE levels in the soluble cortex, frontal cortex, hippocampus, and cerebellum fractions for mice dosed chronically over 8 weeks with vehicle, 2 g/kg oDGal or 4 g/kg oDGal. Cortex (*F*_(2, 18)_ = 10.58, *p =* 0.0009) and cerebellum (*F*_(2, 26)_ = 7.7, *p* = 0.0024) analyzed by one-way ANOVA followed by Dunnett’s multiple comparison test. FC (*F*_(2, 14.49)_ = 5.27, *p* = 0.019) analyzed by Brown–Forsythe and Welch ANOVA followed by Dunnett’s T3 multiple comparison test, *** *p* < 0.001, ** *p* < 0.01. (**B**) AGE levels in the insoluble cortex, frontal cortex, hippocampus, and cerebellum fractions for mice dosed chronically over 8 weeks with vehicle, 2 g/kg oDGal or 4 g/kg oDGal. Data are presented as a mean ± SEM percentage vehicle. Cortex (*F*_(2, 18)_ = 3.72, *p* = 0.046) and hippocampus (*F*_(2, 26)_ = 10.04, *p* = 0.0006) analyzed by one-way ANOVA followed by Dunnett’s multiple comparison test, * *p* < 0.05; ** *p* < 0.005. (**C**) PC levels in soluble fractions of cortex, frontal cortex, hippocampus, and cerebellum for mice dosed chronically over 8 weeks with vehicle, 2 g/kg oDGal or 4 g/kg oDGal. Data are presented as a mean ± SEM percentage vehicle. Cortex (*F*_(2, 18)_ = 11.85, *p* = 0.0005), hippocampus (*F*_(2, 26)_ = 10.03, *p* = 0.0005), and FC (*F*_(2, 26)_ = 9.174, *p* = 0.001) analyzed by one-way ANOVA followed by Dunnett’s multiple comparison test, * *p* < 0.05, ** *p* < 0.005, *** *p* < 0.0005. (**D**) Representation of BACE1 activity measured in cortex, frontal cortex, hippocampus, and cerebellum homogenates for mice dosed chronically over 8 weeks with vehicle, 2 g/kg oDGal or 4 g/kg oDGal. Data are presented as a mean ± SEM percentage vehicle. Cortex (*F*_(2, 18)_ = 8.94, *p* = 0.002), FC (*F*_(2, 22)_ = 9.5, *p* = 0.001) and hippocampus (*F*_(*2, 26*)_ *=* 7.1, *p = 0.0035*) analyzed by one-way ANOVA followed by Dunnett’s multiple comparison test. Asterix (*) represent significant changes: * *p* < 0.05, ** *p* < 0.005, *** *p* < 0.001. All data are presented as a mean ± SEM percentage vehicle.

Following 8 weeks of oral dosing, both oDGal groups (2 g/kg or 4 g/kg oDGal) exhibited a dose-dependent increase in soluble AGE in the cortex and frontal cortex (Figure 2A). Elevations in insoluble AGE were also observed in the cortex (Figure 2B). No change in hippocampal soluble AGE levels was evident for either oDGal dose group relative to vehicle. There was, however, a significant increase in insoluble AGE in the hippocampus of the 4 g/kg oDGal group (Figure 2B). Only soluble AGE levels were elevated in the cerebellum of the 4 g/kg oDGal group (Figure 2A), while insoluble AGE levels were undetectable. In addition to exerting effects on AGE levels, oDGal treatment dose-dependently elevated protein carbonyl (PC) levels, a measure of ROS, in the cortex, frontal cortex, and hippocampus (Figure 2C). oDGal did not affect PC levels in the cerebellum, and a similar lack of oxidative damage in the cerebellum has been reported in the clinic for sAD patients [30,31,32]. An increase in the activity (Figure 2D) of BACE1 in the cortex, frontal cortex, and hippocampus of oDGal treated animals was also noted.

### 2.3. oDGal Increases Aβ and Hyperphosphorylated Tau

**Figure 3:**
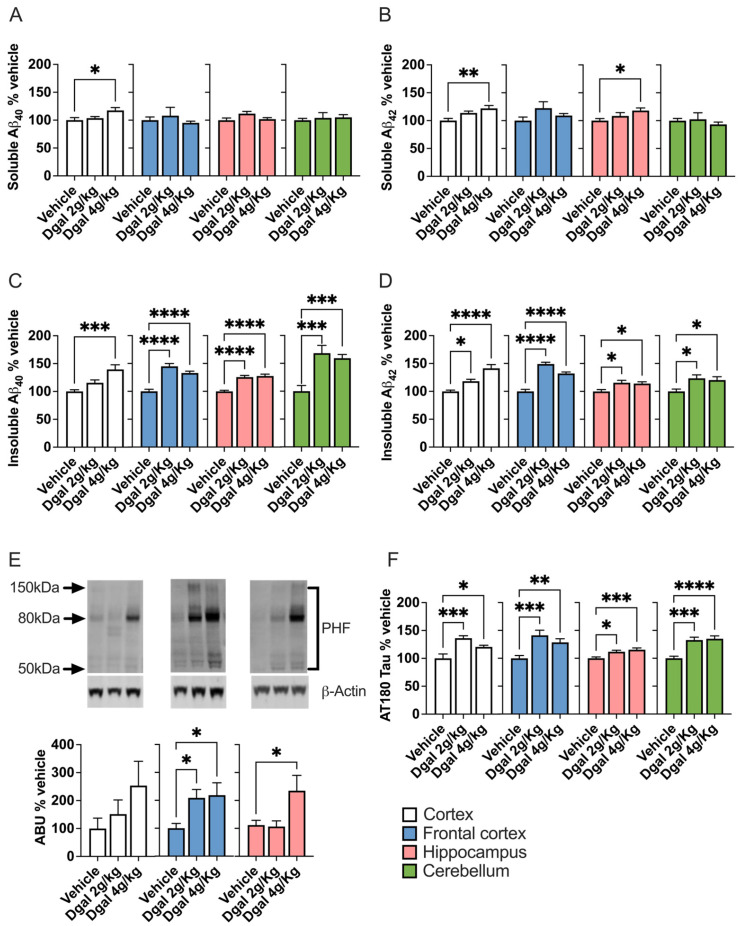
Chronic oDGal treatment promotes Aβ and hyperphosphorylated tau accumulation. (**A**) Soluble Aβ_40_ in the cortex, frontal cortex, hippocampus, and cerebellar fractions of mice dosed chronically over 8 weeks with vehicle, 2 g/kg oDGal or 4 g/kg oDGal. Cortex (*F*_(2, 18)_ = 4.5, *p* = 0.03) analyzed by one-way ANOVA followed by Dunnett’s multiple comparison test, * *p* < 0.05. (**B**) Soluble Aβ_42_ in the cortex, frontal cortex, hippocampus, and cerebellar fractions of mice dosed chronically over 8 weeks with vehicle, 2 g/kg oDGal or 4 g/kg oDGal. Cortex (*F*_(2, 18)_ = 6.2, *p* = 0.009) and hippocampus (*F*_(2, 26)_ = 3.853, *p* = 0.045) analyzed by one-way ANOVA followed by Dunnett’s multiple comparison test, * *p* < 0.05, ** *p* < 0.005. (**C**) Insoluble Aβ_40_ in the cortex, frontal cortex, hippocampus, and cerebellar fractions of mice dosed chronically over 8 weeks with vehicle, 2 g/kg oDGal or 4 g/kg oDGal. Cortex (*F*_(2, 18)_ = 9.553, *p* = 0.0015), FC (*F*_(2, 26)_ = 33.16, *p*< 0.0001), hippocampus (*F*_(2, 26)_ = 33.26, *p*< 0.0001), and cerebellum (*F*_(2, 24)_ = 14, *p* < 0.0001) analyzed by one-way ANOVA followed by Dunnett’s multiple comparison test, *** *p* < 0.001, **** *p* < 0.0001. (**D**) Insoluble Aβ_42_ in the cortex, frontal cortex, hippocampus, and cerebellar fractions of mice dosed chronically over 8 weeks with vehicle, 2 g/kg oDGal or 4 g/kg oDGal. Cortex (*F*_(2, 18)_ = 18.75, *p* < 0.0001), FC (*F*_(2, 24)_ = 57.29, *p* < 0.0001), hippocampus (*F*_(2, 26)_ = 6.24, *p* = 0.007), and cerebellum (*F*_(2, 26)_ = 5.9, *p* = 0.008) analyzed by one-way ANOVA followed by Dunnett’s multiple comparison test, * *p* < 0.05, **** *p* < 0.0001. (**E**) Representative blots and quantification plot for p-tau protein levels detected using AT180, pThr231, antibody. Mice were dosed chronically over 8 weeks with vehicle, 2 g/kg oDGal or 4 g/kg oDGal. Data were normalized to β-actin. FC (*p* = 0.017) analyzed by Kruskil–Wallis test followed by Dunn’s multiple comparison test. Hippocampus (*F*_(2, 26)_ = 4.75, *p* = 0.018) and Cerebellum (*F*_(2, 26)_ = 5.9, *p* = 0.008) analyzed by one-way ANOVA followed by Dunnett’s multiple comparison test. * *p* < 0.05. (**F**) Representation of insoluble p-tau detected using AT180 antibody-coupled MSD Elisa plates for mice dosed chronically over 8 weeks with vehicle, 2 g/kg oDGal or 4 g/kg oDGal. Cortex (*F*_(2, 18)_ = 12, *p* = 0.0005), FC (*F*_(2, 24)_ = 10.13, *p* = 0.0006), hippocampus (*F*_(2, 26)_ = 8.95, *p* = 0.0012), and cerebellum (*F*_(2, 26)_ = 18.95, *p* < 0.0001) analyzed by one-way ANOVA followed by Dunnett’s multiple comparison test. Asterix (*) represent significant changes: * *p* < 0.05, ** *p* < 0.01, *** *p* < 0.001, **** *p* < 0.0001. All data are presented as a mean ± SEM percentage vehicle.

Given our noted modulation of BACE1 activity with oDGal treatment, we next assessed beta amyloid (Aβ) levels in response to these dosing regimens. Aβ was measured in two fractions of brain tissue extract: a soluble fraction (Figure 3A,B) and an insoluble fraction (Figure 3C,D). Following oDGal dosing, the majority of Aβ_40_ and Aβ_42_ accumulated in the insoluble fraction. oDGal treatment significantly increases both insoluble Aβ_40_ and Aβ_42_ levels in the cortex, frontal cortex, hippocampus, and cerebellum. Interestingly, the cortex displays a significant increase in soluble Aβ_40_ and Aβ_42_ in the 4 g/kg oDGal group. Significantly elevated soluble Aβ_42_ was also detected in the hippocampi of the 4 g/kg oDGal group. In addition to Aβ accumulation, the generation of hyperphosphorylated tau is also considered a hallmark of AD pathology. We found that oDGal treatment induced the accumulation of phosphorylated-tau (p-tau) in both soluble (Figure 3E) and insoluble fractions (Figure 3F). Soluble p-tau antibody exposure revealed a broad immunoreactive band extending from 55 kDa to 150 kDa for both oDGal groups in all brain regions except the cerebellum (Figure 3E). This 55 kDa to 150 kDa p-tau immunoreactive band was quantified and plotted in Figure 3E. Similar data have been reported for postmortem AD brain, and this p-tau immunoreactive band (55–150 kDa) is thought to indicate hyperphosphorylated tau aggregation or crosslinking [33].

### 2.4. oDGal Promotes Neuroinflammation

**Figure 4:**
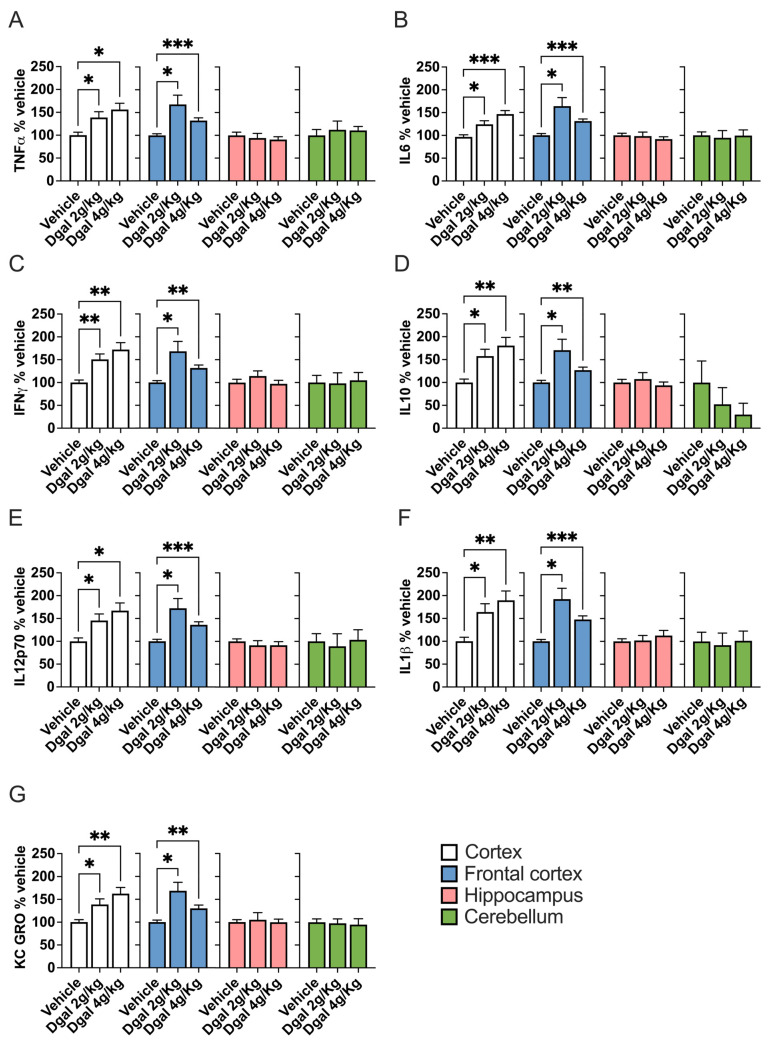
Chronic oDGal dosing elevates inflammatory mediators in the brain. (**A**) TNFα in the soluble cortex, frontal cortex, and hippocampal fractions for mice dosed chronically over 8 weeks with vehicle, 2 g/kg oDGal or 4 g/kg oDGal. Cortex (*F*_(2, 14.3)_ = 5.95, *p* = 0.013) and FC (*F*_(2, 8.9)_ = 7.65, *p* = 0.012) analyzed by Brown–Forsythe and Welch ANOVA followed by Dunnett’s T3 multiple comparison test, * *p* < 0.05, *** *p* < 0.001. (**B**) IL6 in the soluble cortex, frontal cortex, and hippocampal fractions for mice dosed chronically over 8 weeks with vehicle, 2 g/kg oDGal or 4 g/kg oDGal. Cortex (*F*_(2, 14.69)_ = 11.72, *p* = 0.0009) and FC (*F*_(2, 8.51)_ = 7.84, *p* = 0.012) analyzed by Brown–Forsy the and Welch ANOVA followed by Dunnett’s T3 multiple comparison test, * *p* < 0.05, *** *p* < 0.001. (**C**) IFNγ in the soluble cortex, frontal cortex and hippocampal fractions for mice dosed chronically over 8 weeks with vehicle, 2 g/kg oDGal or 4 g/kg oDGal. Cortex (*F*_(2, 12.94)_ = 9.532, *p* = 0.0029) and FC (*F*_(2, 9.04)_ = 6.748, *p* = 0.016) analyzed by Brown–Forsythe and Welch ANOVA followed by Dunnett’s T3 multiple comparison test, * *p* < 0.05, ** *p* < 0.01. (**D**) IL10 in the soluble cortex, frontal cortex, and hippocampal fractions for mice dosed chronically over 8 weeks with vehicle, 2 g/kg oDGal or 4 g/kg oDGal. Cortex (*F*_(2, 13.69)_ = 8.02, *p* = 0.0049) and FC (*F*_(2, 8.93)_ = 6.1, *p* = 0.021) analyzed by Brown–Forsythe and Welch ANOVA followed by Dunnett’s T3 multiple comparison test, * *p* < 0.05, ** *p* < 0.01. (**E**) IL12p70 in the soluble cortex, frontal cortex, and hippocampal fractions for mice dosed chronically over 8 weeks with vehicle, 2 g/kg oDGal or 4 g/kg oDGal. Cortex (*F*_(2, 13.88)_ = 6.17, *p* = 0.012) and FC (*F*_(2, 9.28)_ = 7.79, *p* = 0.01) analyzed by Brown–Forsythe and Welch ANOVA followed by Dunnett’s T3 multiple comparison test, * *p* < 0.05, *** *p* < 0.001. (**F**) IL1β in the soluble cortex, frontal cortex, and hippocampal fractions for mice dosed chronically over 8 weeks with vehicle, 2 g/kg oDGal or 4 g/kg oDGal. Cortex (*F*_(2, 13.98)_ = 7.25, *p* = 0.007) and FC (*F*_(2, 9.3)_ = 10.24, *p* = 0.0045) analyzed by Brown–Forsythe and Welch ANOVA followed by Dunnett’s T3 multiple comparison test, * *p* < 0.05, ** *p* < 0.01, *** *p* < 0.001. (**G**) KC GRO in the soluble cortex, frontal cortex, and hippocampal fractions for mice dosed chronically over 8 weeks with vehicle, 2 g/kg oDGal or 4 g/kg oDGal. Cortex (*F*_(2, 13.52)_ = 7.8, *p* = 0.0056) and FC (*F*_(2, 10.42)_ = 8.57, *p* = 0.0063) analyzed by Brown–Forsythe and Welch ANOVA followed by Dunnett’s T3 multiple comparison test. Asterix (*) represent significant changes: * *p* < 0.05, ** *p* < 0.01. All data are presented as a mean ± SEM percentage vehicle.

Chronic inflammation is both a hallmark of AD [34,35] as well as the normal aging process (i.e., inflammaging) [36,37]. We noted a considerable inflammatory response in the oDGal treatment groups—the frontal cortex and cortex—displayed the majority of the inflammatory marker response (Figure 4A–G). Intriguingly, the hippocampus and cerebellum seemed to be spared from significant neuroinflammation at the 8 week time point.

### 2.5. oDGal Affects Multiple Cognitive Domains

**Figure 5:**
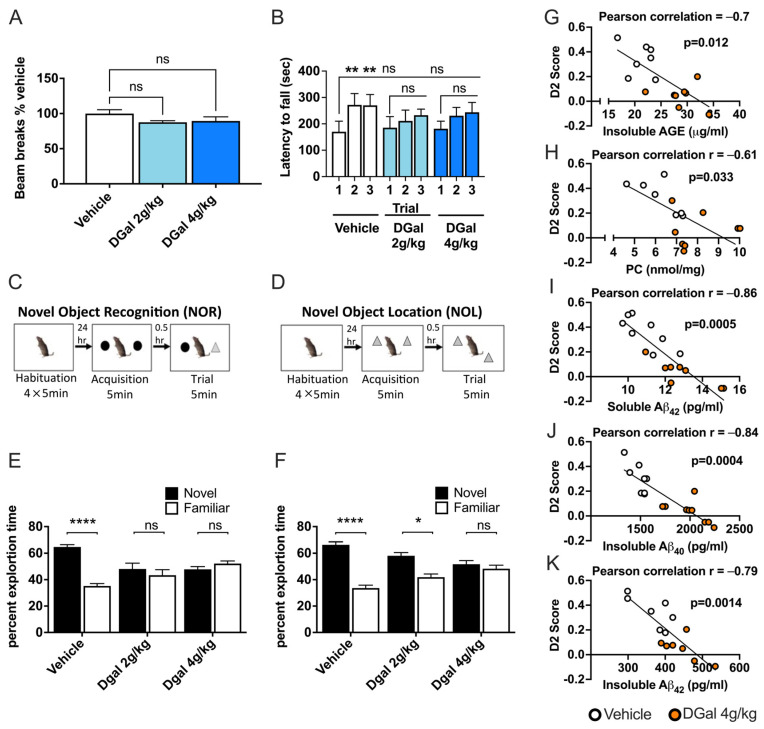
oDGal treatment promotes multi-domain cognitive deficits without affecting motor coordination or activity. (**A**) Average locomotor activity represented by number of beam breaks over a one-hour period for mice dosed chronically over 8 weeks with vehicle, 2 g/kgoDGal, or 4 g/kgoDGal. Data are presented as a percentage of the vehicle group. Data are presented as mean ± SEM, analyzed by one-way ANOVA followed by Dunnett’s multiple comparison test. (**B**) Latency for animal to fall from an accelerating rotarod. Mice were dosed chronically over 8 weeks with vehicle, 2 g/kgoDGal or 4 g/kgoDGal. Data were analyzed using a two-way repeated measures mixed model approach, with treatment factor treatment (*F*_(6, 58)_ = 0.28, *p* = 0.95), repeated factor trial (*F*_(2, 116)_ = 19.20, *p* < 0.001). Schematic representation of NOR (**C**) and NOL (**D**) tasks, where circles and squares represent objects used for the study. (**E**) NOR task represented as a percentage exploration time of familiar and novel objects for mice dosed chronically over 8 weeks with vehicle, 2 g/kgoDGal or 4 g/kgoDGal. Data were analyzed by Kruskil–Wallis test (*p* = 0.0001) followed by Dunnett’s multiple comparison test. (**F**) NOL task represented as a percentage exploration time of familiar and novel locations for mice dosed chronically over 8 weeks with vehicle, 2 g/kgoDGal or 4 g/kgoDGal. Data were analyzed by Brown–Forsythe and Welch ANOVA (*F*_(5, 25.96)_ = 29.99, *p* = 0.0001) followed by Dunnett’s T3 multiple comparison test. Discrimination index (D2) score, calculated from the hippocampal spatial cognitive score, correlated significantly with levels of hippocampal insoluble AGE (**G**), PC (**H**), soluble Aβ_42_ (**I**), insoluble Aβ_40_ (**J**) or insoluble Aβ_42_ (**K**) in mice dosed chronically over 8 weeks with 4 g/kgoDGal or vehicle (Veh), determined by Pearson’s rank correlation coefficient (r). Asterix (*) represent significant changes: * *p* < 0.05, ** *p* < 0.01, **** *p* < 0.0001, ns=non-significant. All data are presented as a mean ± SEM percentage vehicle.

We have demonstrated so far that oDGal oral dosing can result in increased brain Aβ and hyperphosphorylated tau, as well as significant increases in inflammatory mediator expression, all factors that are reminiscent of clinical AD pathology. We next assessed whether these effects of oDGal dosing also caused the creation of cognitive deficits that are highly characteristic in AD patients. With respect to generalized central neurological behavior, oDGal treatment showed no effect on basal activity (Figure 5A) or motor coordination (Figure 5B, trial 1). The vehicle group showed an increased latency to fall from trial 1 to trials 2 and 3 as a result of motor learning. This was not apparent for either oDGal group, suggesting impaired motor learning (Figure 5B). Novel Object Recognition (NOR) (Figure 5C), a memory task dependent on perirhinal cortex functionality, and Novel Object Location (NOL) (Figure 5D), a hippocampal-reliant memory task [38], can be used to evaluate an animal’s ability to recognize a novel object or location, respectively. Vehicle-treated animals (i.e., control) showed a significant preference for the novel object; in contrast, animal groups treated with oDGal failed to demonstrate any recognition or bias for interest in the novel object. These results suggest that oDGal resulted in a potential functional deficit in perirhinal cortex functionality (Figure 5E). Both the vehicle and 2 g/kgoDGal groups showed a preference for the novel location, whereas the 4 g/kgoDGal dosing group failed to notice the novel location (Figure 5F), implying that 4 k/kgoDGal treatment resulted in a hippocampal-dependent cognitive deficit. Indeed, the discrimination index D2 score displays a dose-dependent effect for oDGal in the NOL test but not the NOR test (Figure S1A,B). The ability of 2 g/kgoDGal treatment to affect performance in the NOR test but not the NOL test suggests that the perirhinal cortex is more sensitive than the hippocampus to the oDGal insult (Figure 5E,F).

### 2.6. oDGal-Induced AGE, ROS, and Aβ Modulation Correlate with Spatial Memory Deficits

Hippocampal insoluble AGE (Figure 5G) and PC levels (Figure 5H) correlated significantly with the performance scores in the hippocampal NOL task for the 4 g/kgoDGal group. Soluble hippocampal Aβ_42_ also showed a significant correlation with the hippocampal-dependent spatial NOL task (Figure 5I). Contrary to this, soluble hippocampal Aβ_40_ levels failed to correlate with the NOL performance of oDGal-treated mice. The increase in insoluble Aβ_40_ and Aβ_42_ in the hippocampus of the 4 g/kgoDGal group (Figure 3C,D) also showed a significant correlation with the NOL performance score (Figure 5J,K), implying that in addition to insoluble AGE, PC, and soluble Aβ_42_, both insoluble Aβ_40_ and Aβ_42_ also may have a negative impact on spatial memory. No other pathologies showed any correlation with spatial memory at this time point.

### 2.7. Etiology of oDGal Pathology

**Figure 6:**
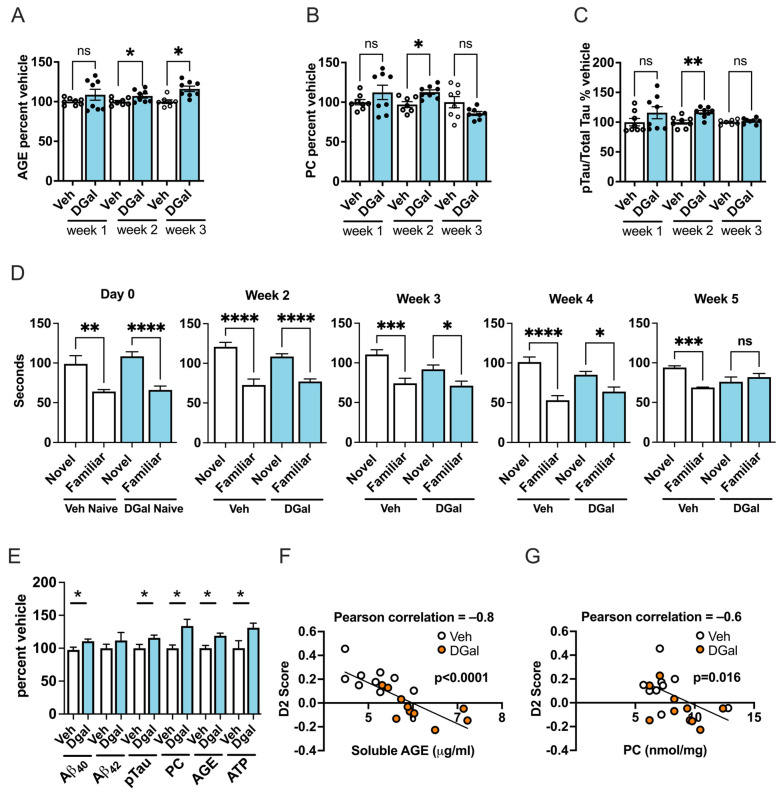
AD-like pathologies precipitate in the hippocampus ahead of other brain regions. AGE (**A**), PCs (**B**), and p-tau (**C**) levels in the soluble hippocampal fraction for mice dosed chronically over 1, 2 or 3 weeks with vehicle (Veh) or 4 g/kgoDGal. Hyperphosphorylated tau was detected using AT180 antibody-coupled MSD ELISA plates and normalized to total tau levels. In (**A**) AGE data are analyzed by one-way ANOVA (*F*_(5, 41)_ = 3.34, *p* = 0.013) followed by Dunnett’s multiple comparison test and unpaired student *t*-test; (**B**) PC (*F*_(5, 20.83)_ = 3.66, *p* = 0.016) and (**C**) p-tau (*F*_(5, 18.66)_ = 4.47, *p* = 0.0076) data are analyzed by Brown–Forsythe and Welch ANOVA followed by Dunnett’s T3 multiple comparison test. (**D**) Spatial recognition test (SRT) represented as exploration time, in seconds, of familiar and novel locations for vehicle (Veh) or 4 g/kgoDGal. Animals were dosed chronically for periods depicted in the figures. Data were analyzed by one-way ANOVA; Day 0: (*F*_(3, 36)_ = 13.49, *p* < 0.0001), week 2: (*F*_(3, 34)_ = 24.22, *p* < 0.0001), week 3: (*F*_(3, 36)_ = 8.47, *p* = 0.0002), week 4: (*F*_(3, 44)_ = 14.68, *p* < 0.0001), and week 5: (*F*_(3, 28)_ = 7.18, *p* = 0.001) followed by Dunnett’s multiple comparison test. (**E**) Representation of Aβ_40_, Aβ_42_, p-Tau, PC, AGE, and ATP detected in the soluble hippocampal fraction from animals dosed chronically over 5 weeks with vehicle (Veh) or 4 g/kgoDGal. Data were analyzed by unpaired Mann–Whitney nonparametric *t*-test. Asterix (*) represent significant changes: * *p* < 0.05; ** *p* < 0.01, **** *p* < 0.0001, *** *p* < 0.001, ns = non-significant, *n* = 10 vehicle and *n* = 10 oDGal mice. All data are presented as a mean ± SEM percentage vehicle. Discrimination index (D2) score, calculated from the hippocampal SRT, correlated with levels of soluble AGE (**F**) and PC (**G**) detected in the hippocampal fraction of mice dosed chronically for 5 weeks with 4 g/kgoDGal or Veh. D2 score correlates with soluble AGE and PC as determined by Pearson rank correlation coefficient (r). *n* = 10 vehicle and *n* =10 oDGal mice.

We have identified that oDGal treatment can induce AD-related pathological features that correlate to cognitive deficits. To extend our appreciation of the etiopathological actions of oDGal treatment, we assessed the central nervous system (CNS) histopathology post-mortem. Brains of mice treated with vehicle or the 4 g/kgoDGal dosing level were collected after 1, 2, and 3 weeks of treatment. No gross morphological and pathological changes were observed in the cortex, frontal cortex, or cerebellum. In contrast, hippocampal regions displayed some pathology (in soluble tissue extract fractions only), suggesting they are affected ahead of the aforementioned regions. oDGal treatment at weeks 2 and 3 induced a significant elevation in hippocampal AGE levels (Figure 6A), while PCs were significantly elevated at the 2 week time point (Figure 6B). A trend of elevated AGE and PCs was observed after 1 week of treatment, suggesting some animals were more resilient than others to the oDGal treatment insult. A small, significant increase in p-tau but not Aβ, was apparent at the 2 week time point (Figure 6C). CNS tissue cytokine elevation was not detected at these time points, indicating that AGE, ROS, and/or p-tau accumulation were strong candidates for initiating the onset of the pathologies observed following 8 weeks of dosing. Interestingly, the pathologies at the 2 week time point did not affect the oDGal animals’ spatial cognitive test performance score (Figure 6D). The reason for this may be that the pathology is simply not at a sufficient level to yet affect cognition, a process generated at the network interaction level [39,40,41]. However, the initial onset of a spatial cognitive deficit becomes apparent following 5 weeks of oDGal treatment (Figure 6D). To gain an understanding of the type of pathology that drives the cognitive deficit at the 5 week time point, brain pathology was assessed as before. The oDGal group displayed a significant increase in CNS tissue AGE, PC, Aβ, p-tau, and ATP levels (Figure 6E). As noted previously, this pathology was only observed in the soluble fraction of the hippocampus. Despite this full range of pathology, AGE and PCs were the only insults to significantly correlate with the spatial hippocampal cognitive score (Figure 6F,G), suggesting that both AGE and PC accumulation over 5 weeks is sufficient to contribute to the initial spatial cognitive deficit.

### 2.8. Therapeutic Interventions Ameliorate oDGal-Induced Pathology and Cognitive Decline

**Figure 7:**
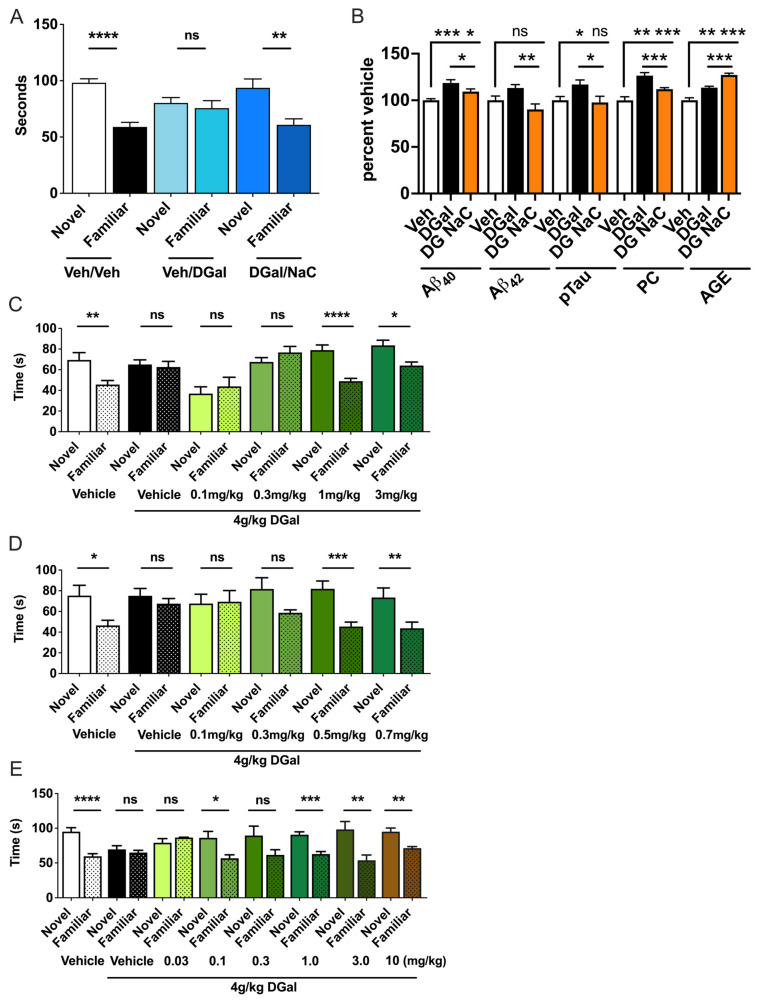
Spatial cognitive deficit and pathology are reversed or delayed pharmacologically. (**A**) SRT is represented as exploration time, in seconds, of familiar and novel locations. Animals were dosed chronically with vehicle or 4 g/kgoDGal with and without 5g/kg NaC for 5 weeks. Data are analyzed by unpaired *t*-test. (**B**) Representation of Aβ_40_, Aβ_42_, p-Tau, PC, and AGE detected in the soluble hippocampal fraction from animals dosed chronically over 5 weeks with vehicle (Veh) or 4 g/kgoDGal with and without 5g/kg NaC for 5 weeks. Data were analyzed by Brown–Forsythe and Welch ANOVA; Aβ_40_: (*F*_(2, 27.7)_ = 11.16, *p* = 0.0005), Aβ_42_: (*F*_(2, 23.65)_ = 5.79, *p* = 0.009), p-Tau: (*F*_(2, 21.79)_ = 3.98, *p* = 0.0036), PC: (*F*_(2, 19.03)_ = 18.18, *p* < 0.0001), and AGE (*F*_(2, 20.91)_ = 44.08, *p* < 0.0001) followed by Dunnett’s T3 multiple comparison test. Hippocampal SRT time spent in novel and familiar area for vehicle and oDGal (4 g/kg) treated mice dosed acutely with memantine (**C**), donepezil (**D**), and leveteracitam (**E**). Mice were treated chronically with vehicle or 4 g/kgoDGal for 5 weeks prior to acute drug treatment. Time spent in novel and familiar location analyzed by paired *t*-test for each treatment. Data are presented as a mean ± SEM. Asterix (*) represent significant changes:* *p* < 0.05, ** *p* < 0.01, *** *p* < 0.001, **** *p* < 0.0001, ns = non-significant. *n* = 10 vehicle and *n* = 10 oDGal mice.

We have contended that ROS are highly likely to contribute to the generation of oDGal-induced AD-like effects. To assess whether the cognitive deficit and/or pathology could be delayed through pharmacological interventions that nullify the toxic effects of ROS, oDGal mice were treated with the antioxidant NaC (N-acetyl cysteine) in combination with oDGal for 5 weeks. The NaC successfully delayed the hippocampal cognitive deficit (Figure 7A) and onset of pathology (Figure 7B) in the oDGal mice in comparison to the oDGal vehicle-treated group. The NaCoDGal group showed a significant reduction in Aβ_40_, Aβ_42_, and hyperphosphorylated tau (Figure 7B). Importantly, the NaCoDGal group also showed a significant reduction in PCs relative to the oDGal vehicle group. The NaCoDGal group did however show a significant increase in AGE in relation to both the vehicle and oDGal vehicle groups (Figure 7B). In order to further validate the oDGal model as an appropriate preclinical model of neurodegeneration, we investigated the ability of actual therapeutic compounds that are approved or in clinical trials for the treatment of AD, donepezil, memantine, and levetiracetam [42,43], to interdict the oDGal-induced cognitive dysfunctions. Animals were dosed with oDGal for 5 weeks before being acutely dosed with donepezil, memantine, or levetiracetam. Importantly, all three compounds reversed the cognitive deficit induced by oDGal treatment (Figure 7C–E). Both donepezil and memantine have been shown to acutely improve learning and memory in transgenic mouse models of AD [44]. Levetiracetam was originally developed as an anti-epileptic drug but has been shown to reverse behavioral deficits in aged wildtype as well as *Tg* AD animal models by reducing Aβ-induced neuronal excitability [45,46]. Thus, taken together, it is evident that oDGal treatment can reproduce many of the pathologies and cognitive deficits characteristic of sAD, and importantly, these AD-promoting effects of oDGal demonstrate a sensitivity to clinically employed dementia interventions. These data therefore support the potential utility of the oDGal murine model in AD drug research.

## 3. Discussion

**Figure 8:**
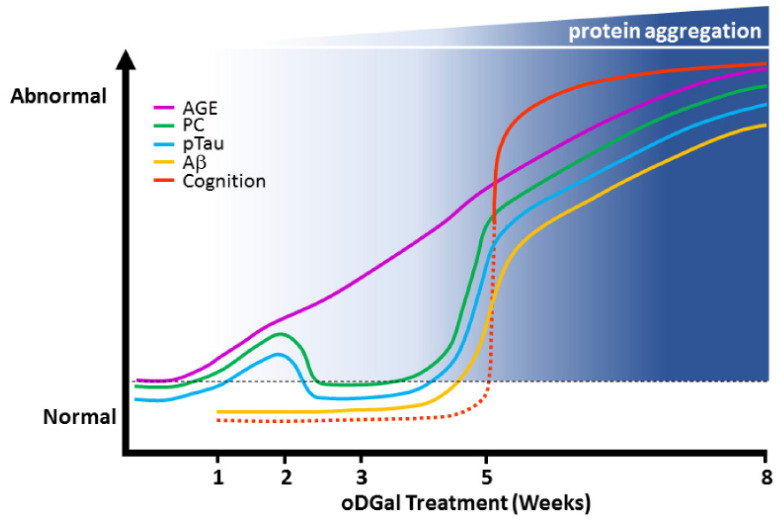
Diagram illustrating sequential onset of pathologies in the hippocampus following oDGal dosing. Spatial cognitive deficit precipitates at 5 weeks and correlates negatively with elevated AGE and PC. AGE, PC, p-tau, and Aβ levels are all elevated in the soluble fraction of the hippocampus following 5 weeks of dosing. At the 8 week time point, these pathologies undergo significant aggregation and propagate to adjacent brain regions such as the cortex, frontal cortex, and cerebellum.

Here we present the characterization of a novel and physiologically relevant oDGal model of sAD. The oDGal model provides an appealing alternative to the traditional *Tg* animals that have been used as preclinical models in AD research to date. We found that our oral-dosing oDGal model exhibited a number of striking features directly relevant to clinical observations of the human disease: (i) oDGal animals display multiple domain cognitive dysfunctions and present with a suite of AD-like pathologies; (ii) Importantly, unlike traditional *Tg* models of AD, the robust, reproducible phenotype and associated pathologies displayed in the oDGal model are not driven by mutant protein expression, and consequently, the etiology observed in this model shares similarities to those perceived to occur in sAD (Figure 8); (iii) as observed in human disease, the oDGal-induced pathologies display key temporal and spatial features that include protein aggregation and propagation (Figure 8); (iv) the cognitive dysfunction, in this model, is reversed with acute application of therapeutics prescribed or trialed to AD patients; and (v) pathology is delayed with NaC treatment, which strongly implicates ROS as the mechanistic driver of downstream pathologies and the associated spatial cognitive impairment. In addition, to these clinically relevant features, the attractiveness of the oDGal model for preclinical research is further elevated given that it is straightforward (via oral administration), inexpensive and rapid to establish. The model can be run in either C57Bl6J or Sev129 mice for pharmacological profiling of novel compounds or biologics. The oDGal model can also be run in *Tg* or knockout mice to determine the impact certain genes have on its neurodegenerative phenotype thereby mitigating the need for any time-consuming interbreeding as is conducted with current *Tg* AD models.

With specific respect to nuances in AD-related pathophysiology, our cognitive data suggested that the perirhinal cortex was affected prior to the hippocampus following oDGal exposure, as animals dosed for 8 weeks with 2 g/kgoDGal were able to perform the hippocampal driven NOL task (Figure 5F) but were impaired in the perirhinal cortex-dependent NOR task after just 2 or 4 weeks of dosing (Figure S2A,B). These data share similarities with clinical observations, where the perirhinal/entorhinal cortex is affected early in the disease course and prior to detrimental alterations occurring at the level of the hippocampus [47,48,49,50].

The pathology displayed in the AD brain is multifaceted, and pathological spreading is a key feature of the disease [51]. *Tg* animal models of AD possess an applied developmental phenotype dependent on the overexpression of mutant proteins, which ultimately affects pathology initiation and progression timing. Although the amyloid cascade hypothesis has been proposed to be responsible for the pathogenesis of fAD, increasing evidence suggests that dysregulated cellular metabolism and oxidative stress play a key role in the etiology of sAD [3,52]. Abnormal levels of oxidative stress markers have been reported in sAD patients [53,54] and precede Aβ accumulation by decades [55,56,57]. Furthermore, FDG-PET imaging data clearly demonstrates significant hypometabolism in brain regions affected by AD decades before the disease may become apparent [58]. Neurometabolic dysfunctions (potentially linked to multiple glycometabolic deficits) are highly likely to result in the exacerbation of oxidative stress as ROS are found to be elevated in pro-diabetic states linked to dementia [59,60,61]. Increased oxidative stress has also been strongly associated with the excessive generation of AGE products. AGE products are induced by protracted exposure to elevated blood glucose, a classical hallmark of pro-diabetic pathology. Therefore, ROS and AGE are likely to both serve as effective markers of AD conditions linked to neurometabolic stress [62,63,64]. In the oDGal model, pathology is detectable 2 weeks post oDGal dosing, with significant changes in ROS, AGE, and p-tau preceding all other pathological insults (Figure 6A–C). Intriguingly, this pathology is present only in the soluble hippocampal fraction. The transient normalization of ROS (Figure 6B) and p-tau (Figure 6C) levels at the 3 week time point may be due to cellular compensation mechanisms initially able to cope with the oDGal insult, prior to their potentially pathological re-appearance at 5 weeks (Figure 6E). Early elevations in p-tau were subtle and reversible, suggesting they were not pathological. p-Tau was found to be elevated at later time points, and by 8 weeks it had spread to other brain regions (Figure 3E,F). By this time, tau is mainly present in the insoluble fraction and has undergone aggregation, typical of the pathologically hyperphosphorylated species. The initial, subtle increase in p-tau indicates early perturbations in tau phosphorylation state and that tau is affected ahead of Aβ in this model. Similarly, intraneuronal tau accumulation has been demonstrated to precede Aβ deposition in human subjects [65,66].

Unlike ROS and p-tau, AGE levels remained abnormally elevated between 2 and 5 weeks of exposure to oDGal (Figure 6A,E). Despite the re-appearance of other pathologies at 5 weeks, including p-tau, ROS, and Aβ, elevated AGE and ROS levels were the only pathologies to negatively correlate with impaired spatial cognition, implicating them as potential drivers of downstream pathologies observed at 5 weeks (Figure 6F,G). AGE levels are elevated in MCI and AD patients and correlate with disease severity [67,68,69,70]. As galactose is metabolized to UDP-galactose, a substrate for AGE, via the Leloir pathway, we hypothesize that the observed elevations in AGE levels in oDGal mice result from activation of this pathway and lead to increased ROS generation. AGE accumulation is linked to accelerated oxidative damage [71], and in-house in vitro data demonstrate that exposure of rodent primary neuronal cultures to AGE-BSA increases the level of nitrotyrosine and 8-oxyguanadine, markers of ROS damage. 

Interestingly, significant evidence indicates the elevation of ROS in mild cognitive impairment (MCI) and sAD, and postmortem autopsy data suggest it may be the earliest pathological feature [31,54,72,73,74,75,76,77,78,79,80,81,82]. ROS has been shown to affect Aβ production through modulating α, γ, and β-secretase [83,84,85]. Elevated BACE1 activity is observed in the AD brain, particularly in areas most susceptible to degeneration [38,39,40]. These changes are observed in areas shown to exhibit significant elevations in protein carbonylation. Thus, the increased BACE1 activity (Figure 2D) observed in the oDGal model may be a consequence of elevated oxidative stress, as suggested by increased PC levels (Figure 2C).

Despite early hippocampal pathologies, spatial cognitive deficits are only apparent after 5 weeks of oDGal dosing (Figure 6D). While pathology at 5 weeks remained spatially restricted to the hippocampus, a pathological spread to other brain regions, including the cortex, had occurred by 8 weeks (Figure 3A–F). Protein misfolding and aggregation, a hallmark of AD [5], are recapitulated in the oDGal model. At early time points, all pathology is detected only in the soluble fraction, contrary to the 8 week time point, where the majority of pathology predominantly presents in the insoluble fraction. This shift from soluble- to insoluble-state pathology indicates a degree of protein misfolding and/or aggregation has occurred over time. Interestingly, AGE have also been shown to chemically crosslink proteins, and, in so doing, act as a seed for plaques in protein aggregating neurodegenerative diseases [86,87,88,89,90,91,92,93].

At the 8 week time point, the spatial cognitive deficit remains in oDGal animals (Figure 5F), which still correlates negatively to ROS and, in this instance, insoluble AGE due to its aggregating nature (Figure 5G,H). In addition to these pathologies, there is also a negative correlation between hippocampal soluble Aβ_42_ and insoluble Aβ_40_ and Aβ_42_ with spatial memory (Figure 5I–K), implicating the aggregated Aβ species as being more toxic.

Phenotypic reversal through pharmacological manipulation is essential when developing novel neurodegeneration models. Importantly, the reversal of the oDGal-induced cognitive deficit was achieved using compounds prescribed/trialed for dementia treatment. A single dose of either donepezil, memantine, or levetiracetam was able to reverse the spatial cognitive deficit elicited by 5 weeks of oDGal dosing (Figure 7C–E). Donepezil is a selective acetylcholinesterase inhibitor widely prescribed for AD. It has shown efficacy in the clinic for mild, moderate, and severe stages of AD. Memantine was designed as a selective NMDA-receptor (NMDAR) antagonist intended to reduce excitotoxicity in moderate- to late-stage AD. The paradox of acute dosing of memantine being able to improve learning and memory is due to its ability to reduce the activation threshold for neuronal depolarization through NMDAR antagonism. Levetiracetam is predicted to enhance cognitive performance using the same principle. Neuronal background ‘noise’ is reduced through normalization of intracellular calcium levels. Reduced glutamate release was detected in isolated hippocampal and cortical synaptosomes from oDGal-treated animals (Figure S3A,B), a factor that may contribute to their reduced cognitive performance. The ability of memantine and levetiracitam to lower the threshold of neuronal activation suggests this is the probable mechanism through which these compounds are eliciting their beneficial effects, although alternative mechanisms cannot be discounted.

To demonstrate that the pathology could be delayed pharmacologically, oDGal mice were treated prophylactically with the antioxidant NaC. NaC has shown efficacy in the clinic; nutraceuticals, a formulation containing NaC delayed cognitive decline and improved the mood of MCI patients. Although it is not clear from this data whether NaC alone is responsible for the improvement, it does suggest that antioxidant treatment may be a beneficial treatment for sAD. Historically, the failure of antioxidants in clinical trials for sAD may have been due to enrolling patients who already displayed symptoms of dementia, where the pathology was too severe to rescue. The NaCoDGal mice displayed a spatial cognitive score comparable to the vehicle-treated animals, demonstrating a complete delay in any spatial cognitive deficit, contrary to what was seen in the oDGal group (Figure 7A). The NaCoDGal group also showed a significant reduction in all pathologies measured, Aβ_40_, Aβ_42_, pTau, and importantly, PC levels relative to the oDGal group (Figure 7B). Surprisingly, the NaCoDGal group showed a significant increase in AGE levels relative to both the vehicle and the oDGal group, despite previously correlating negatively with spatial cognition (Figure 6F). The elevated AGE and lack of cognitive impairment in the oDGalNaC group imply that ROS is responsible for the cognitive deficit and downstream pathologies. The fact that AGE showed a significant negative correlation with spatial memory indicates that AGE is triggering ROS production, as demonstrated by other groups [71,94] and our own in-house data. Although the phenotypic rescue of the oDGal model with NaC strongly implies ROS is involved in our observed pathological phenotypes [95,96,97,98], direct measurement of ROS levels would be a productive new route for further studies.

In summary, the oDGal model is a novel and pragmatic example of sAD, displaying a central phenotype with no obvious peripheral abnormalities (Figure S4A–P). The utility of this model is that it allows the investigation of both prodromal and symptomatic sAD through simple manipulation of dosing, which determines the pathology type and its effects on different cognitive domains. Clinical evidence dictates that early intervention is imperative for successful disease-modifying therapeutics. In contrast to fAD, mutant protein expression does not drive sAD pathology. Therefore, both the type and progression of pathology will differ significantly between these subtypes of disease, particularly during the critical initiation phase, in which pharmacological intervention would be most beneficial. While the etiology of sAD remains unknown, accumulating evidence indicates a crucial role for oxidative stress in the genesis of this disease. Importantly, key temporal and spatial features of oDGal pathology, which correlate with significant cognitive impairment, link to observations made in human disease. We propose that the oDGal model provides an excellent, novel, robust, and facile model in which the initial pathological insults appear similar to those manifesting early on in the clinic and provides an attractive alternative to the traditional *Tg*AD mice to model sAD.

## 4. Materials and Methods

### 4.1. Animals

Male C57Bl6/j mice (Charles River, Harlow, UK) were used throughout the study. Animals were group housed on a 12 h: 12 h light–dark cycle, and food and water were available ad libitum. All procedures described were approved by the Home Office and were designed with a commitment to reduce numbers and undue suffering in accordance with the Animals (Scientific Procedures) Act 1986.

### 4.2. D-Galactose (oDGal) Treatment

D-galactose (2 g/kg or 4 g/kg) and 0.1% sodium benzoate were supplemented into the oDGal group’s drinking water; vehicle (Veh) groups received 0.1% sodium benzoate in their drinking water. Dosing started at three months of age, and drinking water was changed every second day using freshly prepared stock. Animals could access their food and drinking water ad libitum, and this was their only water source throughout the experiment.

### 4.3. oDGal Formulation

A 150 g/L D-galactose (Sigma, Gillingham, UK, G0625) stock solution was made in drinking water. To generate the 4 g/kgoDGal dosing material, we applied the following protocol: Given a mouse drinks 2 mL of water per day, the animals were given a solution of 2 mg/g/mL (for 30 g mice), hence created with 60 mg/mL of D-galactose. The 2 g/kg received a drinking water solution of half this concentration. The final oDGal formulation was supplemented with 0.1% sodium benzoate (Sigma, Gillingham, UK, 71,300). All experimental groups, Veh and oDGal, received 0.1% sodium benzoate in their drinking water.

### 4.4. Synaptosome Preparation

Hippocampal and cortical synaptosomes were isolated from mice pretreated with vehicle or 4 g/kg oDGal for 8 weeks. Dissected tissue was homogenized in 320 mM sucrose buffer before being centrifuged at 3020× *g*/2 min/4 °C. Supernatants (S1) were centrifuged at 14,600× *g*/12 min/4 °C, and pellets (P2) were re-suspended in 320 mM sucrose buffer before being loaded onto Percoll gradients (3, 10, and 23% from top to bottom, 4 °C). The synaptosomal fraction accumulated between the nominal 10% and 23% Percoll layers following a 35,100× *g*/6min/4 °C centrifugation. The synaptosomal fraction was carefully removed and re-suspended in HEPES buffer (140 mM NaCl, 5 mM KCl, 5 mM NaHCO_3_, 1.2 mM NaH_2_PO_4_, 1 mM MgCl_2_, 10 mM glucose, and 10 mM HEPES, pH 7.6, 4 °C) before being centrifuged at 27,000× *g*/10 min/4 °C. Pellets were re-suspended in HEPES buffer, and the synaptosomal concentration was determined using the BCA assay (Bio-Rad, Watford, UK, 23221). The required aliquots of synaptosomal proteins were finally centrifuged at 3020× *g*/10 min/4 °C. Supernatants were removed and pellets stored at 4 °C until use in the experiment.

### 4.5. Mitochondrial Functional Assay

Mitochondrial function was measured using the Seahorse extracellular flux (XF) 96 analyzer (Seahorse Bioscience, Agilent, Santa Clara, CA95051, USA). On the day of the experiment, XF-PS plates were coated with poly-D-lysine (50 μg/mL) for 2 h and washed with cell culture grade water. 50 mg of synaptosomes were plated on poly-D-lysine-coated XF-PS plates in an ionic medium (20 mM HEPES, 10 mM glucose, 1.2 mM Na_2_HPO_4_, 1 mM MgCl_2_, 5 mM NaHCO_3_, 5 mM KCl, and 140 mM NaCl). The plate was centrifuged at 3400× *g* for 1 h at 4 °C. The ionic medium was replaced with incubation medium (3.5 mM KCl, 120 mM NaCl, 1.3 mM CaCl_2_, 0.4 mM KH_2_PO_4_, 1.2 mM Na_2_SO_4_, 2 mM MgSO_4_, 15 mM Glucose, and 4mg/ml BSA). The plate was incubated and loaded onto the Seahorse XF96 analyzer following the manufacturer’s instructions. All experiments were performed at 37 °C. Titrations determined the optimal concentrations of oligomycin (160 μM), carbonyl cyanide-p-trifluoromethoxyphenyl-hydrazon (FCCP) (14 μM), rotenone (4 μM), and antimycin A (4 μM). By sequentially adding these, we measured basal respiration and ATP production.

### 4.6. Tissue Processing

Frozen sections were homogenized in phosphate buffered saline (PBS) supplemented with complete protease inhibitors (Roche, Welwyn Garden City, UK, 04693116001): 200 μL for the hippocampus and frontal cortex, 400 μL for the cortex and cerebellum, using the LT Tissuelyser (Qiagen, Manchester, UK). An equivalent weight/volume of homogenizing buffer was used for the peripheral tissues. Protein quantification was carried out using the BCA assay, and samples were normalized to 5 mg/mL total protein in PBS containing 1% lauryl-β-D-maltoside. Samples were briefly mixed and left on ice for 30 min before undergoing centrifugation at 16,000× *g*/20 min/4 °C. The supernatants (soluble fraction) were removed, and the remaining pellets were briefly sonicated in Guanidine buffer (6 M guanidine-HCL, 1% sarkosyl, 50 mM Tris, pH 8) before being rotated overnight at room temperature. Guanidine samples were diluted 40× in PBS prior to use (insoluble fraction).

### 4.7. ATP Assay

The Abcam ATP assay kit (Abcam, Cambridge, UK, ab83355) was used to measure ATP levels in 20 μg of soluble fraction, and the manufacturer’s protocols were followed.

### 4.8. Protein Carbonyl and AGE Quantification

Protein carbonyls and AGE products were quantified using the oxiselect protein carbonyl ELISA kit (2B Scientific, Stonesfield, UK, STA-310) and oxiselect AGE ELISA kit (2B Scientific, Stonesfield, UK, STA-317). Plates were incubated with 10 μg/mL of soluble or insoluble fraction at 4 °C overnight, and the manufacturers’ protocols were followed.

### 4.9. BACE1 Activity Assessment

BACE1 activity assay plates (Abcam, Cambridge, UK, ab65357) were loaded with 20 μg of soluble fraction, and the manufacturer’s protocols were followed.

### 4.10. Mesoscale Discovery (MSD) ELISA

Aβ was quantified using the Aβ MSD Vplex kit (Rockville, MD 20850-3173, USA, K15199E), 25 μL of soluble or insoluble fraction were loaded for each brain region. Total and phosphorylated Tau was quantified using a Phospho(Thr231)/Total Tau Kit (Rockville, MD 20850-3173, USA, K15121D). Soluble samples were diluted 80× in PBS, and previously 40× diluted insoluble samples were used. Cytokines were quantified using the Mouse ProInflammatory 7-Plex Tissue Culture Kit (Rockville, MD 20850-3173, USA, K15012B). Soluble fraction samples were diluted 5× in PBS.

### 4.11. Western Blot Analysis

Lysates were resolved using one-dimensional gel electrophoresis on 4–12% gradient acrylamide gels followed by electro transfer to polyvinylenedifluoride (PVDF) (Life technologies, Renfrew, UK, 88520). PVDF membranes were blocked for one hour at room temperature in 4% BSA (Sigma) before application of specific primary antisera. Secondary, species-specific antibodies were used at a dilution of 1:10,000 (Li-COR Bioscience, Lincoln, NE, USA, VRDye 490, 549), and signals were detected with an Odyssey Scanner (Li-COR Bioscience, Lincoln, NE, USA). Specific primary antisera used were obtained from the following sources: AT180 (Pierce, Oxford, UK, MN1040); β-actin (Sigma, Gillingham, UK, A2228).

### 4.12. Locomotor Activity Assay (LMA)

Activity was measured using the PAS Home Cage and Open Field system (San Diego Instruments, San Diego, CA, USA). Mice were individually placed in the LMA boxes, and their activity was recorded for over one hour.

### 4.13. Rotarod Analyses

Mice were placed upon an accelerating rotarod (Ugo Basile), and their latency to fall was recorded. Each mouse received three trials of 10 min each, with an intermittent trial interval of 30 min. Motor coordination was assessed by comparing the latency to fall on the very first trial between treatment groups [99,100]. Motor learning was assessed both within and between subjects by comparing the latency to fall from the first trial with that of subsequent trials [101,102,103].

### 4.14. Novel Object Recognition (NOR)

On day 1, the animals were habituated in the arena with their cage mates for 10 min. The animals were then individually habituated in the arena for 5 min on days 2, 3, and 4. On day 5, the animals were exposed to a sample phase of four identical objects in the arena for 5 min before being returned to their home cage; these would serve as the familiar objects. Following a 30 min intermittent time interval, animals underwent a test phase where they were exposed to two novel objects alongside two familiar objects. The animals were allowed to investigate for 5 min, and the time spent investigating objects was recorded and analyzed manually in a blinded fashion. In order to ensure there was no object bias, each group was split into two, with each sub-group receiving a different set of objects during the sample phase. These sub-groups were further divided in two during the test phase, where the novel objects were placed either on the left or right of the familiar objects. A detailed description of the equipment can be found in the supporting information.

### 4.15. Novel Object Location (NOL)

Animals underwent the same habituation process as per the NOR protocol. On day 5, animals were exposed to a sample phase where they were exposed to two identical objects placed diagonally from one another in the arena for 5 min before being returned to their home cage; these served as the familiar objects. Following a 30 min intermittent time interval, animals underwent a test phase where they were exposed to the same two objects from the sample phase. One of the objects was placed in a novel location compared to its position during the sample phase. The animals were allowed to investigate for 5 min, and the time spent investigating objects was recorded and analyzed manually in a blinded fashion. In order to ensure there was no location bias, each group was split into two, with each sub-group being presented with a different object location during the sample phase. These sub-groups were further divided in two during the test phase, where the novel locations were alternated within groups. A detailed description of the equipment can be found in the supporting information.

### 4.16. Spatial Recognition/Topographical Test

Spatial/topographical recognition memory was tested using the Spatial/topographical Recognition Test (SRT), which is based on the spontaneous tendency of mice to investigate a novel environment over a familiar environment. The test consists of an initial 5 min sample phase, followed by a retention interval, and subsequently a 5 min choice phase. SRT has a higher throughput than the NOL test and was used as an alternative spatial cognitive assay in experiments that required large cohorts.

Mice were acclimatized to the experimental room for 1 h before being individually placed into the front chamber of the spatial recognition box for the sample phase. Mice were allowed to investigate one compartment for 5 min, whilst the other was blocked off, before being returned to their home cage for the retention interval. The retention interval was 3 h; previous experiments indicate that 3 h is the maximum time after which naïve animals can still remember their previous exposure to this task. Sample phase compartment sides were randomly assigned and balanced across each group. Following the retention interval, mice were then placed back into the SRT apparatus for the choice phase, in which access to both compartments was allowed. A detailed description of the equipment can be found in the supporting information. Spatial recognition memory was examined by recording the time spent actively exploring either the novel or familiar compartments. A discrimination index was calculated as follows: Discrimination index = (Time spent exploring novel compartment − time spent exploring familiar compartment)/(Total time spent in novel and familiar compartments).

### 4.17. Drug Treatments

Donepezil-HCl (1224981), memantine-HCl (1380502), and levetiracetam (PHR1447) were purchased from Sigma-Aldrich (Gillingham, UK), and they were all dissolved in 0.9% saline. Mice received a single intraperitoneal (i.p.) injection at a volume of 10 mL/kg of body weight of either donepezil-HCl (0.1, 0.3, 0.5, or 0.7 mg/kg), memantine-HCl (0.1, 1, 3, 5, or 10 mg/kg), levetiracetam (0.1, 0.3, 1, 3, or 10 mg/kg), or vehicle (0.9% saline). Mice were dosed 30 min prior to the sample phase of the SRT. N-acetyl cysteine (NaC) (5 g/kg) (Sigma, Gillingham, UK, 106425) was dissolved in drinking water and pH adjusted to 7.4 with sodium hydroxide. Treatment started on the same day as oDGal and lasted for the duration of the experiment.

### 4.18. Statistics

All data are presented as mean ± 1 SEM. Analyses were performed using GraphPad Prism (GraphPad Software, LLC, version 8.3.0, San Diego, CA, USA) or InVivostat (v4.4.0). Parametric data was analyzed using one-way analysis of variance (ANOVA) or Brown-Forsythe and Welch ANOVA followed by post-hoc comparisons with Dunnett’s, Dunnette’s T3, Sidak’s or Bonferonni multiple comparison tests where appropriate. Nonparametric data was analyzed using Kruskal-Wallis one-way ANOVA followed by Dunn’s multiple comparisons test. An effect was considered significant if *p* < 0.05.

## Data Availability

Data is contained within the article or supplementary material.

## Supplementary Materials

The following supporting information can be downloaded at: https://www.mdpi.com/article/10.3390/ijms24086953/s1.

## Abbreviations

oDGal: oral D-galactose; AD, Alzheimer’s disease; sAD, Sporadic Alzheimer’s disease; fAD, familial AD; *Tg,* transgenic; NaC, N-acetyl-cysteine; ROS, reactive oxygen species, STZ, streptozotocin; Aβ, amyloid beta; AGE, Advanced glycation end-products; NOR, Novel Object Recognition; NOL, Novel Object Location; PC, protein carbonyl; p-tau, hyperphosphorylated tau; SRT, spatial recognition test; D2, Discrimination index; LMA, Locomotor Activity Assay; MSD, Mesoscale Discovery; PVDF, polyvinylenedifluoride; PBS, phosphate buffered saline.
