# Supplementary material for: The oDGal Mouse: A Novel, Physiologically Relevant Rodent Model of Sporadic Alzheimer’s Disease
Source: Int J Mol Sci. 2023 Apr 9;24(8):6953. doi: 10.3390/ijms24086953 (PMC10138655; doi:10.3390/ijms24086953)
Supplement: Supplementary file 1 [file ijms-24-06953-s001.zip › ijms-2291234-supplementary file.pdf]

SUPPLEMENTARY MATERIALS

Supplementary Figures

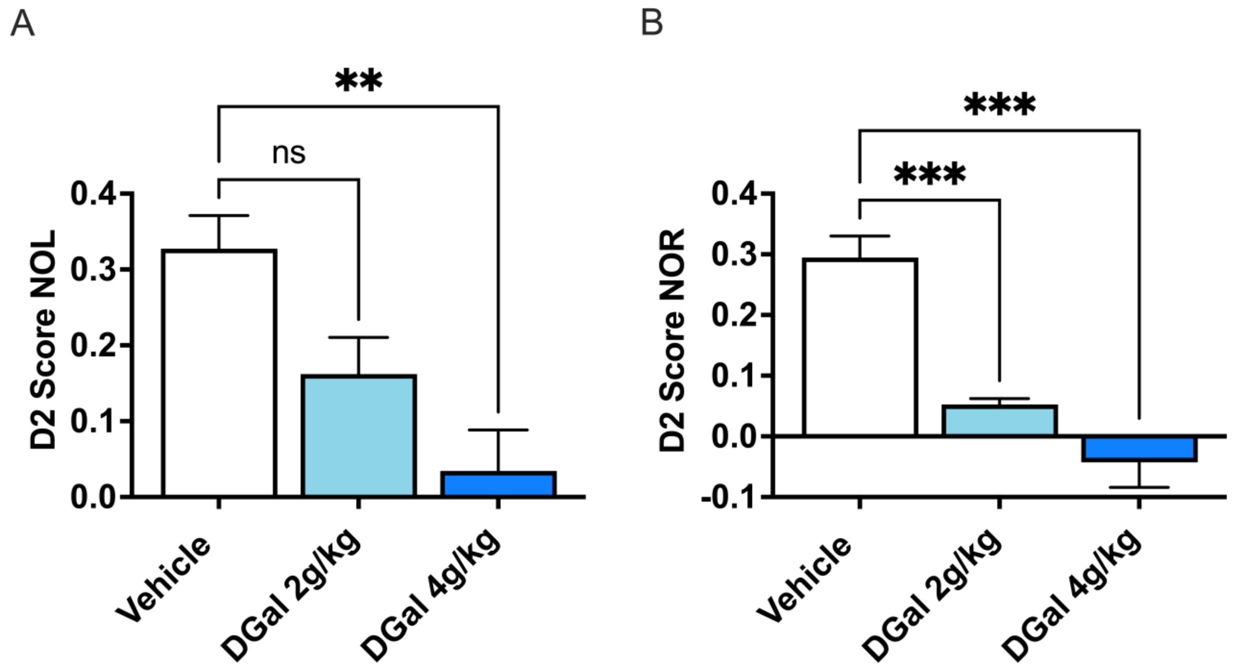

Supplementary Figure S1. NOL and NOR discrimination index (D2) score. NOL (A) and NOR (B) discrimination index (D2) score for mice dosed chronically over 8 weeks with 2g/kg oDGal, 4g/kg oDGal or vehicle. D2 calculation: subtract novel object exploration time from familiar object exploration time, then divide this by the total exploration time. Data analyzed by Brown-Forsythe and Welch ANOVA; NOL *($F_{(2,12.35)}$ = 10.68, p=0.002)*, NOR *($F_{(2,10.33)}$ = 33.86, p<0.0001)* followed by Dunnett's T3 multiple comparison test. Data are presented as a mean ± SEM, **p<0.01, ***p<0.001. n=10 vehicle, n=10 2g/kg oDGal, and n=10 4g/kg oDGal mice.

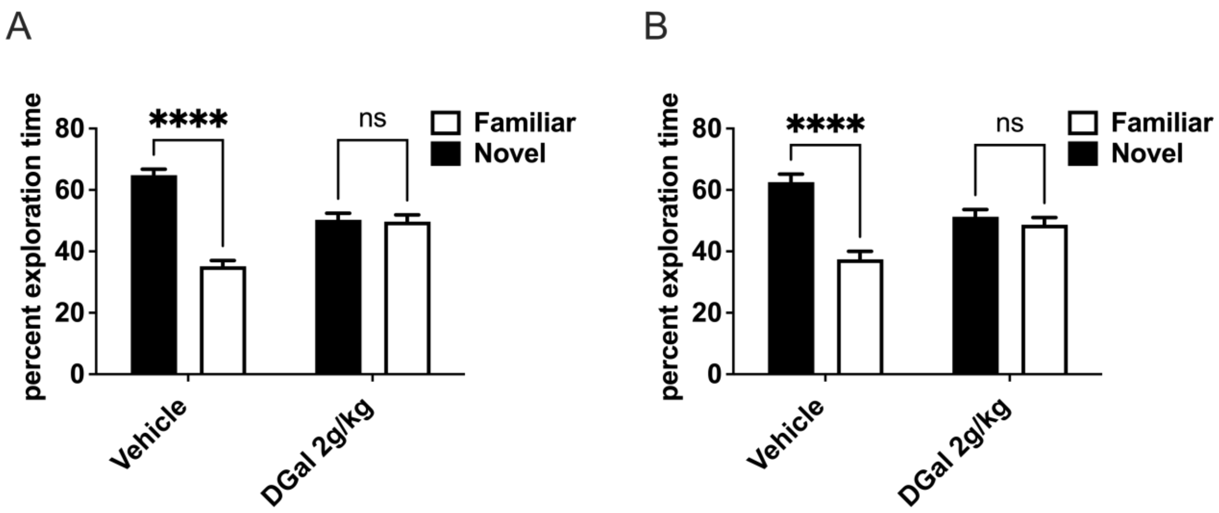

Supplementary Figure S2. Novel object recognition task. Novel object recognition task represented as a percentage exploration time of familiar and novel objects for mice dosed chronically over 2 weeks (A) *($F_{(1,\ 36)}$ = 51,46, $p<0.0001$)* and 4 weeks (B) *($F_{(1,\ 36)}$ = 20.74, $p<0.0001$)* with vehicle or 2g/kg oDGal. Data analyzed by two-way ANOVA followed by Sidak's multiple comparison test. Data are presented as mean ±SEM, ****p<0.01, n=10 vehicle and n=10 oDGal mice.

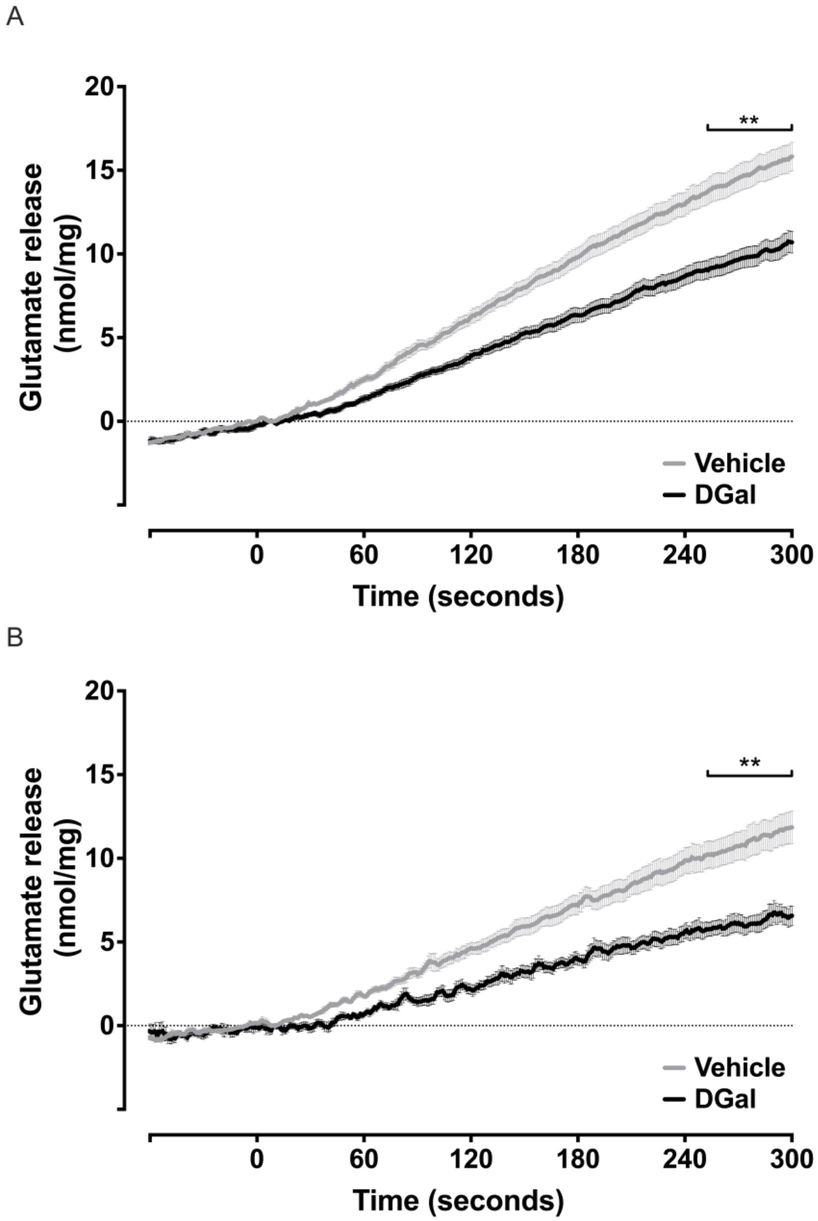

Supplementary Figure S3. Synaptosome glutamate release. 4-Aminopyridine stimulated glutamate release from hippocampal (A) and cortical (B) synaptosomes isolated from mice dosed chronically over 8 weeks with vehicle or 4g/kg oDGal. Data are presented as a mean ± SEM, two tailed t-test, **p<0.01; n=10 vehicle and n=10 oDGal mice.

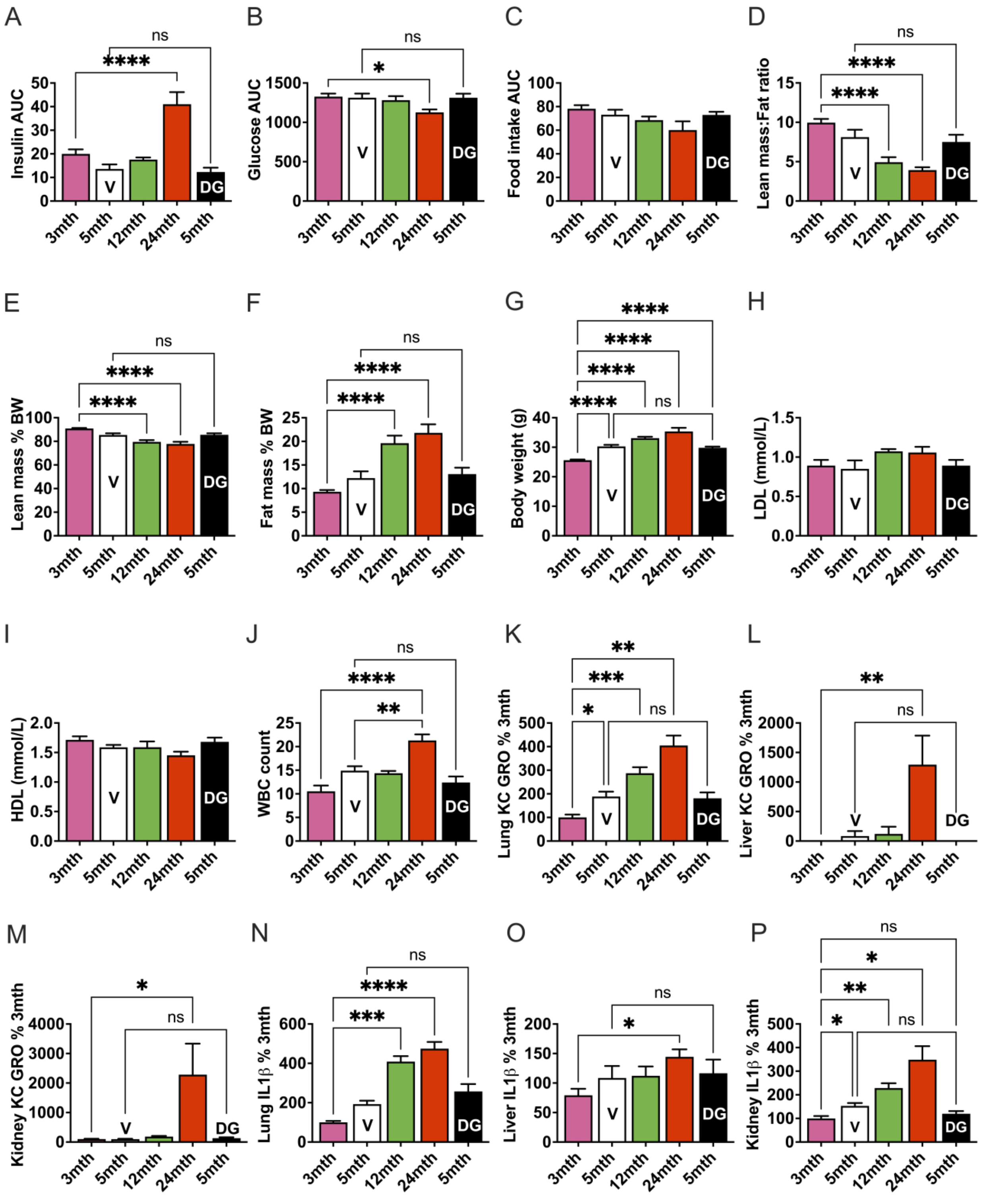

A
Insulin AUC
ns
****
V
DG
3mth
5mth
12mth
24mth
5mth
B
Glucose AUC
ns
*
V
DG
C
Food intake AUC
V
DG
D
Lean mass:Fat ratio
ns
****
****
V
DG
E
Lean mass % BW
ns
****
****
V
DG
F
Fat mass % BW
ns
****
****
V
DG
G
Body weight (g)
****
****
****
****
ns
V
DG
H
LDL (mmol/L)
V
DG
I
HDL (mmol/L)
V
DG
J
WBC count
ns
****
**
V
DG
K
Lung KC GRO % 3mth
**
***
*
ns
V
DG
L
Liver KC GRO % 3mth
**
ns
V
DG
M
Kidney KC GRO % 3mth
*
ns
V
DG
N
Lung IL1β % 3mth
ns
****
***
V
DG
O
Liver IL1β % 3mth
ns
*
V
DG
P
Kidney IL1β % 3mth
ns
*
**
*
ns
V
DG

Supplementary Figure S4. oDGal treatment does not affect peripheral metabolism. (A) Representation of blood insulin levels taken at 0, 10 and 45 minutes following an oGTT in 3, 5, 12 and 24 month old mice. Data are presented as area under the curve (AUC) and analyzed by one-way ANOVA *($F_{(4, 62)} = 19.64$, $p<0.0001$)* followed by Dunnett's multiple comparison test. (B) Oral glucose tolerance test represented as area under the curve (AUC). Glucose tolerance in the mice was measured at multiple time points and is summarized in the graphs by showing AUC for 3, 5, 12 and 24 month old mice. Data analyzed by Brown-Forsythe and Welch ANOVA *($F_{(4, 58.43)} = 2.791$, $p=0.034$)* followed by Dunnett's T3 multiple comparison test. (C) Food intake over 24 hours presented as area under the curve (AUC). Animals were fasted overnight before assessing food intake at 1, 2, 4, 8 and 24 hours. Data analyzed by Brown-Forsythe and Welch ANOVA *($F_{(4, 14.15)} = 2.24$, $p=0.12$)* followed by Dunnett's T3 multiple comparison test. (D) Ratio of whole body lean mass to whole body fat content. Data analyzed by Brown-Forsythe and Welch ANOVA *($F_{(4, 44.82)} = 12.47$, $p<0.0001$)* followed by Dunnett's T3 multiple comparison test. (E) Whole body lean mass content represented as a percentage of body weight (BW) for 3, 5, 12 and 24 month old mice. Data analyzed by Brown-Forsythe and Welch ANOVA *($F_{(4, 51.2)} = 14.11$, $p<0.0001$)* followed by Dunnett's T3 multiple comparison test. (F) Whole body fat content represented as a percentage of body weight (BW) for 3, 5, 12 and 24 month old mice. Data analyzed by Brown-Forsythe and Welch ANOVA *($F_{(4, 52.10)} = 13.72$, $p<0.0001$)* followed by Dunnett's T3 multiple comparison test. (G) Mouse body weight in grams (g) on day of sacrifice. Data analyzed by Brown-Forsythe and Welch ANOVA *($F_{(4, 23.36)} = 28.67$, $p<0.0001$)* followed by Dunnett's T3 multiple comparison test. Serum low density lipoprotein (LDL) *($F_{(4, 37.3)} = 1.97$, $p=0.12$)* (H) and high density lipoprotein (HDL) *($F_{(4, 36.64)} = 2.1$, $p=0.1$)* (I) content for 3, 5, 12 and 24 month old mice. Data analyzed by Brown-Forsythe and Welch ANOVA followed by Dunnett's T3 multiple comparison test. (J) White blood cell (WBC) count was quantified in 3, 5, 12 and 24 month old mice. Data analyzed by Brown-Forsythe and Welch ANOVA *($F_{(4, 47.56)} = 13.98$, $p<0.0001$)* followed by Dunnett's T3 multiple comparison test. KC GRO levels in lung (K), liver (L) and kidney (M) for 3, 5, 12 and 24 month old mice. Lung data analyzed by Brown-Forsythe and Welch ANOVA *($F_{(4, 19.5)} = 18.4$, $p<0.0001$)* followed by Dunnett's T3 multiple comparison test; liver *($F_{(4, 34)} = 5.68$, $p=0.0013$)* and kidney *($F_{(4, 35)} = 4.23$, $p=0.0067$)* data analyzed by one-way ANOVA followed by Dunnett's multiple comparison test. IL1β levels in lung (N), liver (O) and kidney (P) for 3, 5, 12 and 24 month old mice. Lung data analyzed by Kruskil-Wallis test (*$p<0.0001$*) followed by Dunn's

multiple comparison test; liver *($F_{(4, 24.97)} = 3.46, p=0.034$)* and kidney *($F_{(4, 10.47)} = 12.45, p=0.0006$)* data analyzed by one-way ANOVA followed by Dunnett's multiple comparison test. Data are presented as a mean ± SEM, n=12. *p<0.05; **p<0.01, ***p<0.001, ****p<0.0001. Five month old mice were treated chronically with vehicle (V) or 4g/kg oDGal (DG) from 3 months of age for 8 weeks.

Supplementary Methods

NOR/NOL Equipment. The apparatus for the NOR and NOL tasks consisted of a circular arena, containing sawdust, 48.5cm in diameter and 20cm in height made from matt grey 5mm thick Perspex. The arena was placed within a black Perspex box (79cm x 59cm x 68cm) which was open at the front end. A black tarpaulin sheet hung over the opening leaving a 1 inch gap at the bottom. An infrared camera was placed over the arena, inside the box, to record sessions. Objects were placed an equidistance from one another, approximately 8cm from the arena wall. Velcro was used to secure the objects to the base of the arena.

SRT Equipment. The test used a two compartment spatial recognition apparatus (Harvard Apparatus). The box consists of a front chamber made from transparent Perspex™ (20cmx7cmx20cm), which is connected by sliding doors on the left and right to two separate compartments. Both compartments are made from opaque Perspex™ and are of equal surface area (20cmx18cmx20cm). The compartments are differentiated by visual cues (one having walls of black polka dots and the other light grey stripes both on a white background) and room shape (which can be manipulated). The whole apparatus is situated inside a black opaque Perspex™ box (59cmx68cmx79cm) with a camera mounted on the top inner side and an opening on one side for access that contains plastic curtains with an opening of 2cm at the bottom.

Synaptosome Glutamate Release Assay. Glutamate release was assayed using on-line fluorimetry. 0.07mg/ml pelleted synaptosomes were re-suspended in HEPES buffer supplemented with 1mg/ml bovine serum albumin, $NADP^+$ (1mM) and glutamate dehydrogenase (50units/ml). Synaptosome suspensions were transferred to cuvettes and placed inside a Perkin–Elmer LS-55 spectrofluorometer maintained at 37°C. $CaCl_2$ (1mM) was added after 3 minutes. Following 10 minutes of incubation glutamate release was stimulated by the addition of 4-aminopyridine (1

mM). NADPH fluorescence was measured at excitation and emission wavelengths of 340 and 460 nM over 5 minutes. A standard of exogenous glutamate (2.5 nmol) was added at the end of each experiment and the fluorescence change produced by the standard addition used to calculate the released glutamate.

Oral glucose tolerance test (oGTT)

Mice were fasted 16 hours overnight, after which 5μl blood samples were taken via tail tip bleed and basal blood glucose levels determined using a handheld glucometer (OneTouch-Ultra, LifeScan). Animals were then challenged with an oral gavage of 1.5mg/g glucose and blood glucose levels measured via tail tip bleed at 10, 30, 45, 60 and 90 minutes.

Blood insulin

Insulin levels were established by taking whole blood form the tail tip at 0, 10 and 45 minutes and concentrations established by using the Ultra-sensitive mouse insulin ELISA kit (CrystalChem). Area-Under-Curve (AUC) analysis was carried out using GraphPad Prism data analysis package.

Cholesterol

Serum low density lipoprotein (LDL) and high density lipoprotein (HDL) analysis were carried out by Addenbrooke’s Hospital Pathology Department, Cambridge.

Blood count

Full blood count was analyzed using an automated blood count machine (ABC Vet).

MRI body composition

Body composition was established using an automated eMRI system running EchoMRI software.
